# National profile of pediatric emergency medical service utilisation in Saudi Arabia: epidemiological insights for public health and paramedic preparedness

**DOI:** 10.3389/fped.2025.1700334

**Published:** 2026-01-16

**Authors:** Abdulrhman Alghamdi, Rayan Jafnan Alharbi, Abdullah Alshibani, Meshal Alharbi, Ahmed M. Alotaibi, Suliman Ali Alraqebah, Hisham Alqarzaee, Talal Alhafdi, Turki Almubrad, Abdullah Alabdali

**Affiliations:** 1 Emergency Medical Services Department, College of Applied Medical Sciences, King Saud Bin Abdulaziz University for Health Sciences, Riyadh, Saudi Arabia; 2 King Abdullah International Medical Research Center, Riyadh, Saudi Arabia; 3 Emergency Department, King Abdulaziz Medical City, Ministry of National Guard Health Affairs, Riyadh, Saudi Arabia; 4 Emergency Medical Services Program, Department of Nursing, College of Nursing and Health Sciences, Jazan University, Jazan, Saudi Arabia; 5 Central Clinical School, Faculty of Medicine Nursing and Health Sciences, Monash University, Melbourne, VIC, Australia

**Keywords:** emergency medical services, pediatric epidemiology, pediatrics, prehospital care, Saudi Arabia

## Abstract

**Background:**

Emergency Medical Services (EMS) play a vital role in pediatric care by providing timely interventions that can influence outcomes. However, there is limited national evidence from Saudi Arabia describing the profile and patterns of pediatric EMS use. Understanding these patterns is essential to guide paramedic preparedness, system planning, and public health strategies. This study aimed describe the nature of characteristics of emergency calls responded by emergency medical services in Saudi Arabia for pediatric patients aged <18 years during the year of 2022.

**Methods:**

A retrospective, descriptive study was conducted using data from the Saudi Red Crescent Authority (SRCA) for the year 2022. All pediatric cases (<18 years) attended by SRCA were included. Demographic, clinical, temporal, and geographic variables were analysed using descriptive and comparative statistics.

**Results:**

A total of 58,554 pediatric EMS cases were included. Adolescents (47.1%) and children aged 4–12 years (38.9%) formed the largest groups, with a predominance of males (66.2%) and Saudi nationals (81.7%). Most calls were received between 9:00 pm and 1:00 am, with higher frequencies observed during weekends. Regional distribution showed the highest proportions in the Western (37.2%) and Central (30.7%) regions. Among the chief complaint, the most frequent complaints were trauma (21.8%), musculoskeletal (13.3%), bleeding (10.5%), and neurological (10.1%). Most EMS activations were initiated through the 997-call line (79.0%), followed by 911 (11.1%) and mobile applications (9.9%). The majority were managed by basic life support crews (75.1%), while advanced life support attended 22.5% of cases. The median dispatch-to-scene arrival time was 11.71 min, and the median on-scene time was 12.35 min.

**Conclusion:**

This national study provides the first comprehensive profile of pediatric EMS use in Saudi Arabia. The findings highlight the predominance of adolescents and males, the significant burden of medical emergencies alongside trauma, and the broad spectrum of pediatric conditions requiring EMS response. These results emphasise the need to strengthen pediatric-focused training, optimise EMS system planning, and integrate EMS data into public health strategies to improve child health and safety in the Kingdom.

## 1 Introduction

Emergency Medical Services (EMS) plays a vital role in delivering timely interventions that can significantly impact the pediatric patient journey (1). Understanding the most common cases that EMS responds to for pediatric patients is essential for improving resource allocation, paramedic training, and overall prehospital emergency care. Overall, pediatric Emergency Department (ED) visits vary worldwide. For example, in the United States (US), it comprised an estimated 20% of all ED visits (2, 3). In Saudi Arabia, children were reported to comprise 27% of all ED visits in a tertiary hospital in the city of Riyadh (4).

The number of pediatric emergencies requiring EMS activations varied across the globe. It ranges between 5% and 20% of total EMS calls (5, 6). For example, a study in the US shows that pediatric calls represented 7% of EMS activations (6). In addition, comparing the number of pediatric EMS activations before and during the Coronavirus disease 2019 (COVID-19) pandemic showed a decrease number of activations by 28% during the pandemic (1,083 vs. 780 respectively (7). Moreover, the non-transport EMS pediatric calls were shown to be around 30% of all EMS pediatric calls (8, 9). Interestingly, out of the calls that were not transported, which were initiated by paramedics, had low ED attending, and admission within 48 h of EMS activation (10). The pediatric cases are more likely to be non-transportable compared to adult cases (8). This could be because of the possibility of caregiver preference.

The activations of pediatric EMS calls were generated for different causes, such as respiratory and neurological problems. Over the globe, there are variations in the most common causes of calls. For example, in the US, the common diagnoses for pediatric patients transported by EMS, trauma (27%), neurologic (19%), and respiratory (18%) (11). Researchers found that the most common chief complaints in India were fever (22.5%), trauma (21.0%), and respiratory difficulty (14.6%) (12). Consequently, the EMS in low-and middle-income countries had several gaps, including EMS structures and public awareness of EMS (13). In addition, the burden of EMS dealing with pediatric cases in South Africa was the interfacility transportation (14). The majority of EMS activations were low acuity cases (14).

Being prepared to pediatric care in EMS is challenging; therefore, proposed frequent training is essential to maintain the skills and knowledge required during such intense cases. For example, training by using simulation showed improvement in overall EMS personnel performance (15). Therefore, the epidemiology of pediatric EMS use may have important public health implications and can help to guide efforts in both EMS operations and training. This study aims to describe the nature of characteristics of emergency calls responded by emergency medical services in Saudi Arabia for pediatric patients aged <18 years during the year of 2022.

## 2 Material and methods

### 2.1 Study setting and design

This is a retrospective descriptive study of pediatric patients transported by the Saudi Red Crescent Authority (SRCA) between 1 January 2022 to 31 December 2022. Data collected by paramedics of the SRCA for all pediatric cases they responded to in 2022 were retrieved for this study. The SRCA, as the primary provider of prehospital care in Saudi Arabia, is responsible for responding to all emergency cases in the prehospital phase. This includes all kinds of medical and trauma emergencies in addition to mass casualty accidents. The SRCA is also responsible to providing emergency medical care during mass gathering events in Saudi Arabia, such as Hajj and Umrah. Established in 1963, the SRCA now operates 528 centers across the country, providing prehospital care to the entire population (16).

### 2.2 Study population

The study included all pediatric patients who aged <18 years (33), were transported by SRCA during the period from the 1st of January 2022 to the 31st of December 2022, and initiated emergency call through the emergency line in Saudi Arabia (911), SRCA direct line (997), or software applications to request EMS response (Tawakkalna, Sehhaty, and Asefny). The exclusion criteria include call related to pregnancy cases. Furthermore, interfacility transfer cases were excluded as the study aimed to describe the nature of characteristics of emergency calls responded by emergency medical services in Saudi Arabia for pediatric patients aged <18 years during the year of 2022.

We included all patients meeting the inclusion criteria were included in the study. This approach was selected by the investigators as the most appropriate to achieve the study's primary aim to describe the nature of characteristics of emergency calls responded by emergency medical services in Saudi Arabia for pediatric patients aged <18 years during the year of 2022. Therefore, this technique was applied to include all pediatric cases transported by SRCA for the year of 2022. The resulting sample was 58,554 pediatric cases which is sufficient to perform the study analyses.

### 2.3 Data collection and management

Data was collected through the SRCA database for all pediatric cases transported by SRCA during the years of 2022 and met our inclusion criteria. Ethical approval was obtained from King Abdullah International Medical Research Center to conduct this study. The study was conducted in accordance with the declaration of Helsinki. No identifiable information was collected from the study population. To extract the data, An EXCEL sheet was generated to extract the following variables: patient's demographic information (age, sex, and nationality), case-related information [chief complaint, case type [trauma or medical], case location [indoor, outdoor, or unspecified], case geographic region, and case date and time], and SRCA-related information (type of responded crew [advanced or basic life support], call nature [initiated by 911, 977, or software applications], and response time [receiving call to dispatch crew, dispatch to scene arrival, scene arrival to patient, scene time, and call duration]. To clarify, 911 is the emergency line in Saudi Arabia where people living in the kingdom can report any emergency to the police, civil defense, and medical emergency services. This line is run by the national center for security operations under the Ministry of Interior. The line 997 is the direct line for the Saudi Red Cresent Authority to report any medical emergency case in Saudi Arabia. in addition to phone lines, Saudi Arabia has several governmental software applications where people can report any medical emergency case including Tawkkalna, Sehhaty, and Asefny. All these software applications have facilitated the process of reporting and responding to medical emergency cases across the kingdom. All collected data were anatomized and securely stored on a password-protected university desktop, accessible only to the study investigators.

### 2.4 Statistical analysis

Data analyses were conducted using IBM SPSS Statistics 25. Descriptive analyses were performed where frequencies and percentages were reported for categorical variables and mean, and Standard Deviation (SD) were reported for continuous variables. To facilitate interpretation, the findings of the study were expressed using tables and figures. Heatmaps were developed to illustrate the geographic distribution of cases across regions in Saudi Arabia, as well as temporal patterns of emergency calls by hours and days of the week. The average response times across each region in Saudi Arabia was analysed and presented using line charts. All analyses were descriptive in nature and aimed to provide a comprehensive overview of the profile of pediatric EMS use in Saudi Arabia.

## 3 Results

During the study period, SARCA recived 786,035 calls. Out of a total 62,385 pediatric calls, 3,831 records contained missing or incorrect entries and were excluded. The final dataset included 58,554 validated pediatric calls after data quality checks and removal of incomplete records. A total of 58,554 pediatric cases were identified and analyzed. Most cases were commonly related to adolescents (47.12%), male sex (66.22%), and Saudi citizens (81.71%). The majority of cases (65.5%) were medical in nature and occurred indoors (57.14%). The most common reported chief complaint across all age groups was related to trauma (21.84%), followed by musculoskeletal (13.34%) and bleeding (10.51%). In terms of emergency response to these cases, most of them were initiated through SRCA line number 997 (78.98%) and were managed by basic life support crew (75.10%). A summary of the baseline characteristics of these cases is presented in Table 1.

### 3.1 Geographic distribution of the cases in Saudi Arabia

**Figure 1:**
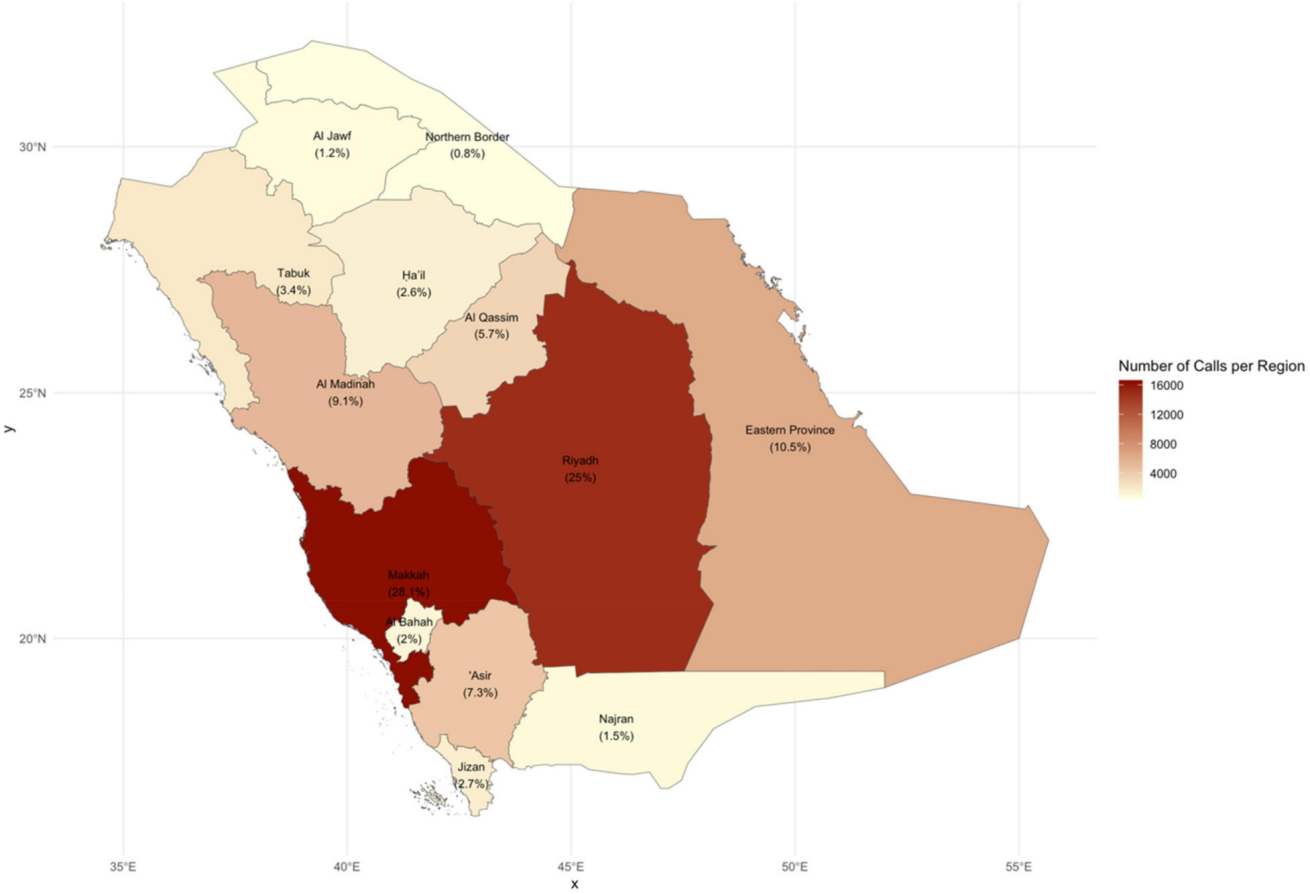
Geographic distribution of reported cases across the provinces of Saudi Arabia.

With regards to cases distribution across the regions of Saudi Arabia, the west (37.18%) and central (30.72%) regions of Saudi Arbia had the proportions of pediatric cases compared to other regions in the kingdom (Tables 1, 2) (34, 35). Furthermore, the heatmap of Saudi Arbia showed that Makkah province in the west region and Riyadh province in the central region of Saudi Arbia had a much higher proportions of pediatric cases compared to the remaining provinces in the kingdom (28.1% and 25%, respectively) (Figure 1).

**Table 1:** Demographic and clinical characteristics of the study population.

Characteristic	*N* (%)
Age	
Infant (0 to ≤ 1 year)	2,793 (4.77)
Toddler (>1 to 3 years)	2,902 (4.96)
Child (>3 to 11 years)	22,177 (37.87)
Adolescent (>11 to <18 years)	30,682 (52.40)
Sex	
Female	19,781 (33.78)
Male	38,773 (66.22)
Nationality	
Saudi	47,846 (81.71)
Non-Saudi	10,708 (18.29)
Case Type based on notification to SRCA	
Medical	38,351 (65.5)
Trauma	20,202 (34.5)
Location	
Indoor	33,455 (57.14)
Outdoor	23,921 (40.85)
Unspecified	1,178 (2.01)
Chief complaint based on response team	
Trauma	12,788 (21.84)
Musculoskeletal	7,814 (13.34)
Bleeding	6,155 (10.51)
Neurological	5,885 (10.05)
General	5,751 (9.82)
Respiratory	5,020 (8.57)
Gastrointestinal	4,500 (7.69)
Dizziness	4,450 (7.59)
Other (e.g., child abuse, congenital desisesetc)	3,302 (5.64)
Cardiac	1,219 (2.08)
Psychiatric	661 (1.13)
Delivery	436 (0.74)
Burn	254 (0.43)
Allergies	207 (0.35)
Abuse	112 (0.19)
Region	
Central	17,988 (30.72)
North	4,743 (8.1)
South	7,908 (13.51)
West	21,772 (37.18)
East	6,143 (10.49)
Call nature	
911	6,489 (11.08)
Phone call (997)	46,247 (78.98)
App (Tawkklna, Aseafni, Sehty, Electronic Moseaf)	5,818 (9.94)
Response crew	
ALS	13,144 (22.45)
BLS	43,973 (75.1)
Unspecified	1,437 (2.45)

SRCA, Saudi Red crescent authority; ALS, advanced life support; BLS, basic life support.

**Table 2:** Population-adjusted rate (PAR).

Region	Total population	Pediatrics	Cases	Rate per 100k/total population	Rate per 100k pediatrics/pediatrics
Riyadh	8,591,748	2,223,209	14,645	170.4543	658.7325
Makkah	8,021,463	2,169,324	16,454	205.1247	758.4851
Maddina	2,137,983	670,168	5,318	248.7391	793.5324
AL-Qassim	1,336,179	411,383	3,343	250.1910	812.6247
Dammam	5,125,254	1,405,054	6,143	119.8575	437.2074
Asir	2,024,285	651,356	4,287	211.7785	658.1654
Tabuk	886,036	299,014	1,987	224.2573	664.5174
Hail	746,406	239,075	1,543	206.7240	645.4042
Arar	373,577	137,756	493	131.9674	357.8791
Jizan	1,404,997	485,730	1,592	113.3099	327.7541
Najran	592,300	214,421	878	148.2357	409.4748
Baha	339,174	110,873	1,151	339.3538	1,038.1247
Jouf	595,822	232,098	720	120.8415	310.2138
Total	32,175,224	9,249,461	58,554		

### 3.2 Cases distribution by time and date

**Figure 2:**
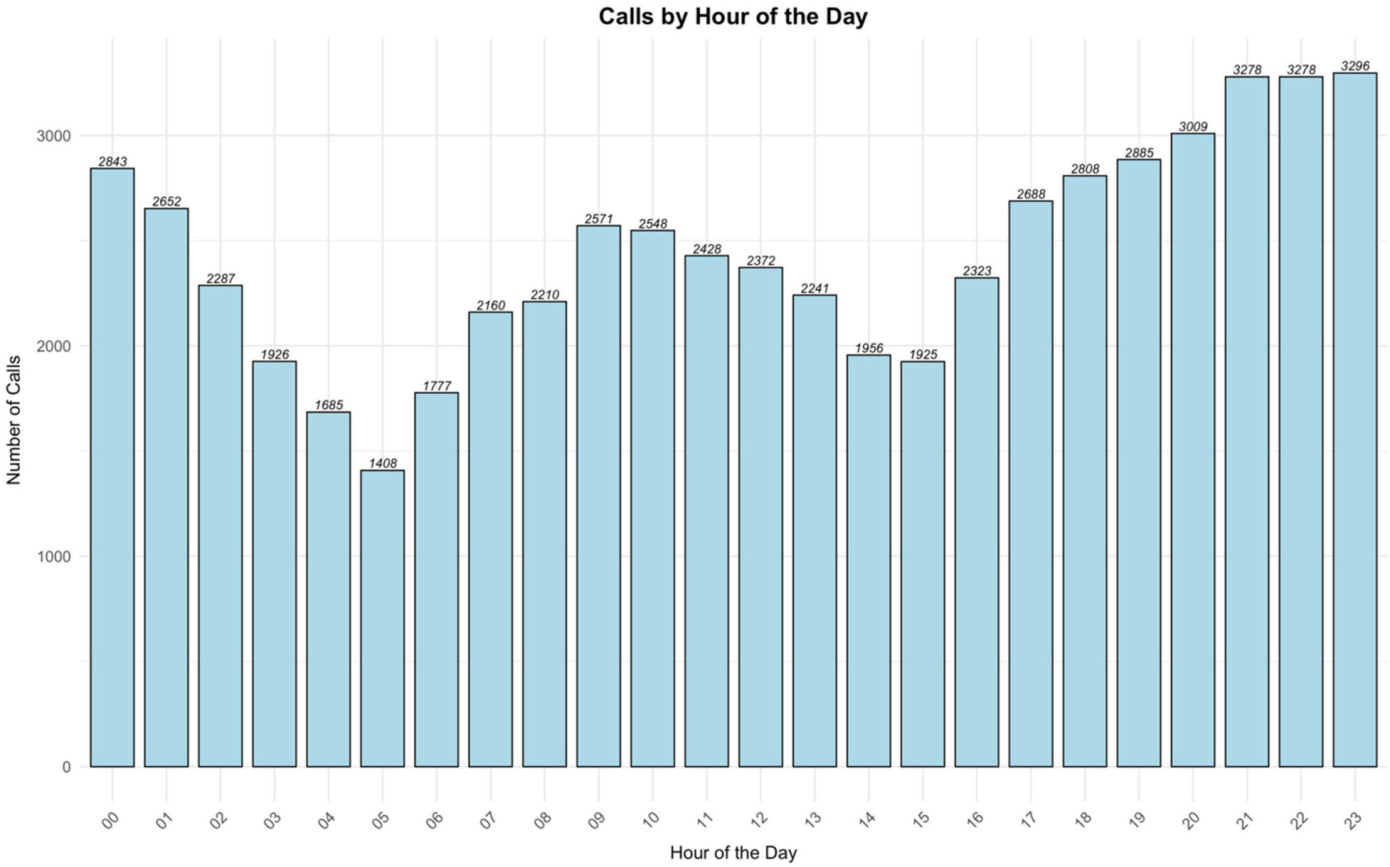
Number of emergency calls by hour of the day.

**Figure 3:**
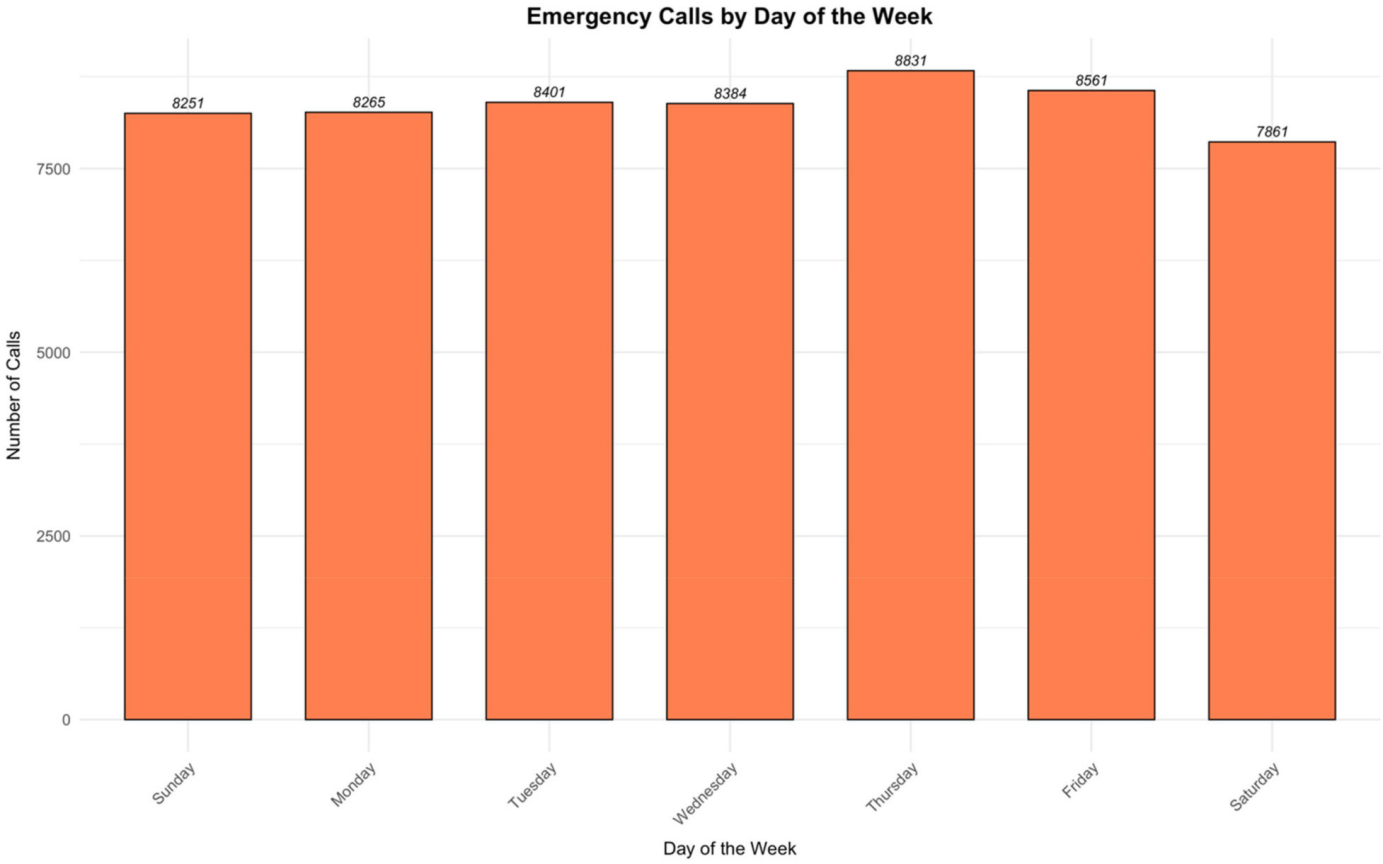
Emergency calls by day of the week.

**Figure 4:**
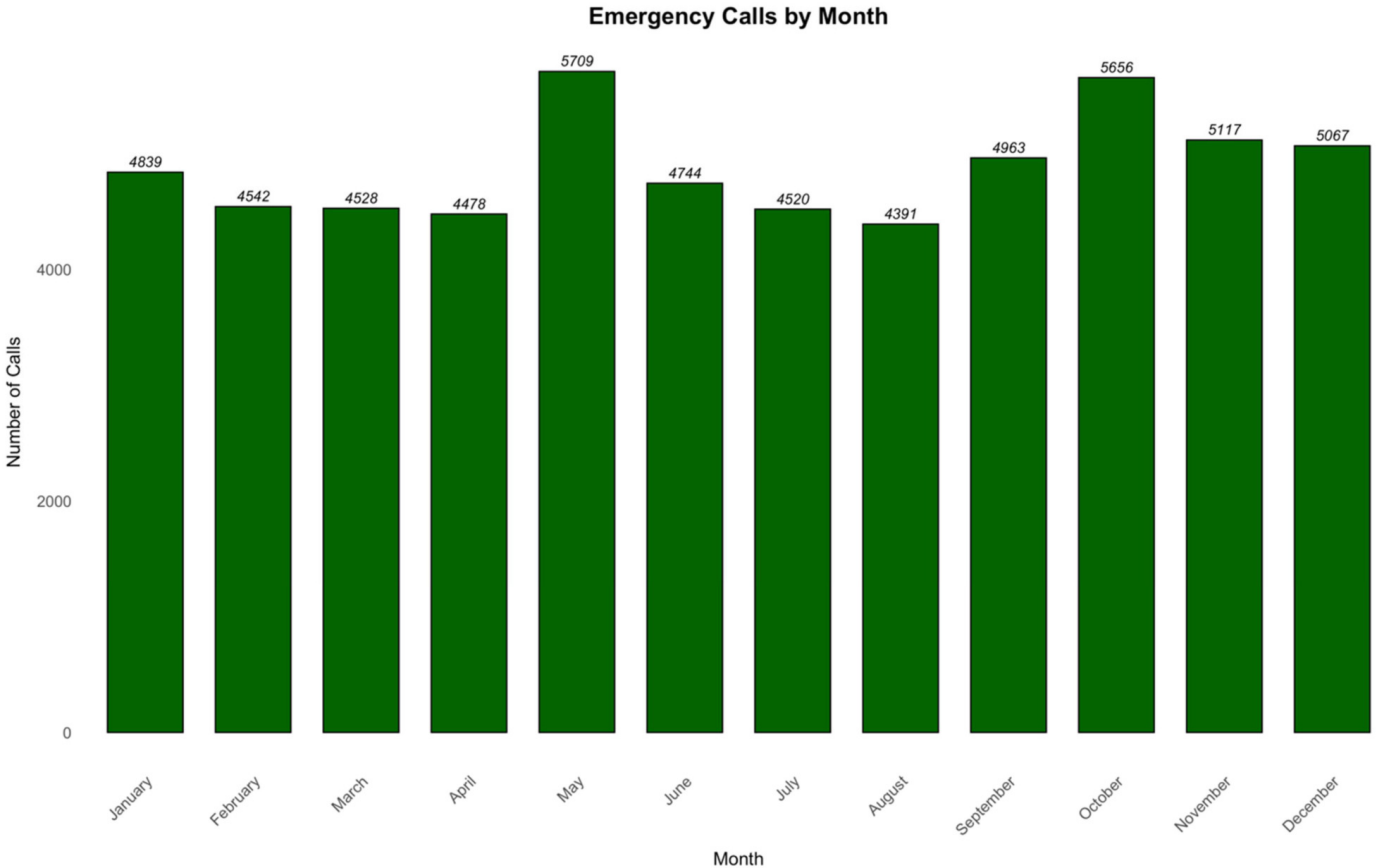
Emergency calls by each month.

**Figure 5:**
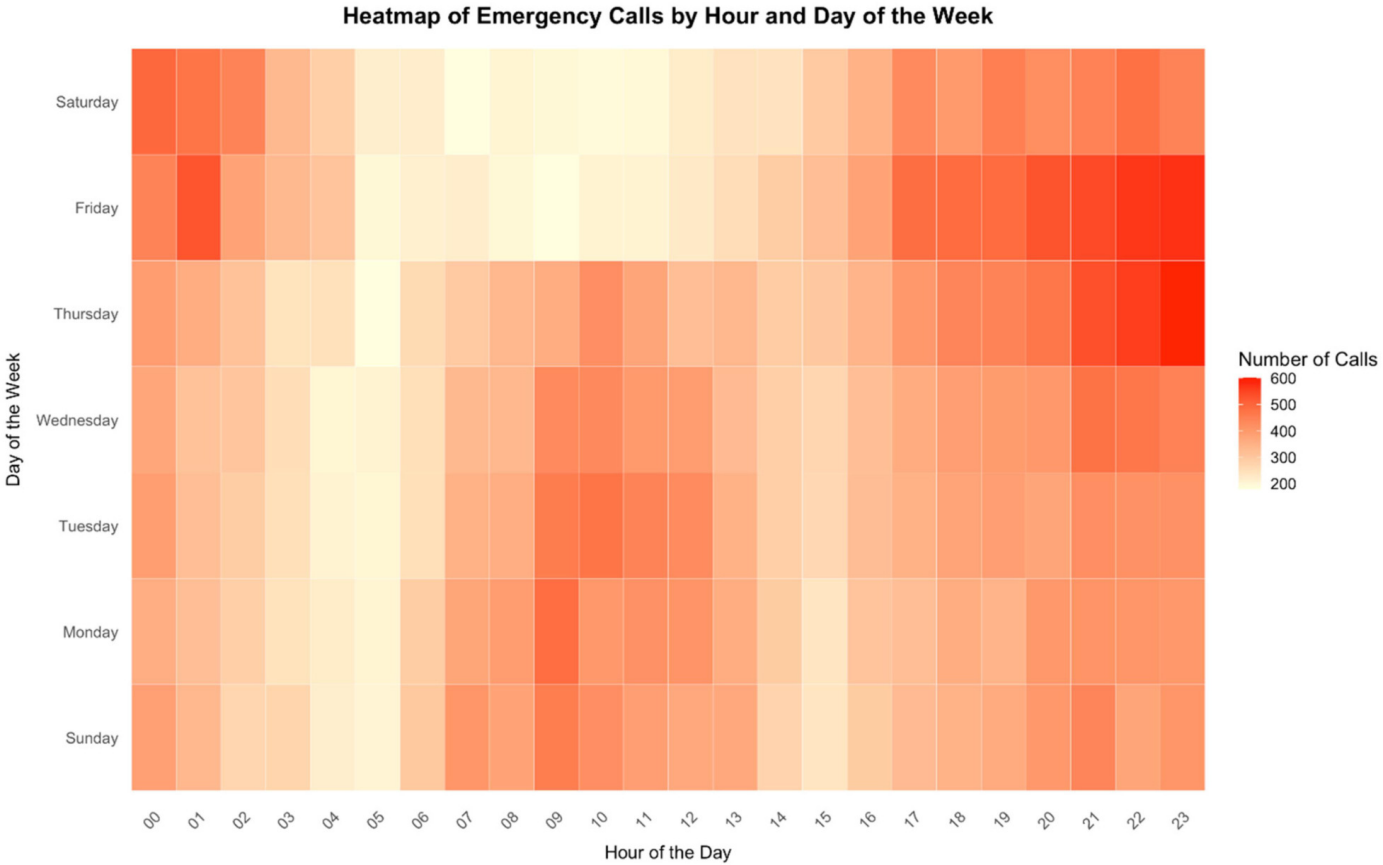
Heatmap of emergency calls by time and day of the week.

Analysis of cases distribution over time revealed that the highest call frequency occurred at 23:00, while the lowest was at 05:00 (Figure 2). Additionally, Thursday recorded the highest number of cases, whereas Saturday was the lowest (Figure 3). On a monthly basis, the highest number of cases reported in May, while August was the lowest (Figure 4). A heatmap of the case hour across the day of the week was developed (Figure 5). During weekends, the heatmap showed that weekend nights (Thursday and Friday nights at 23:00) had the highest proportion of the cases during the weekend and across all times of all days of the week. In contrast, early morning hours to noon (approximately from 05:00 to 13:00) on weekend days (Friday and Saturday) had the lowest proportions of cases (Figure 5). During working days (Sunday to Thursday), most cases were reported around 08:00–09:00 in the morning but were not higher than that reported during weekend nights whereas the lowest proportions of the cases were reported during the early hours of the day from 04:00 to 05:00 (Figure 5).

### 3.3 Response time by EMS

**Figure 6:**
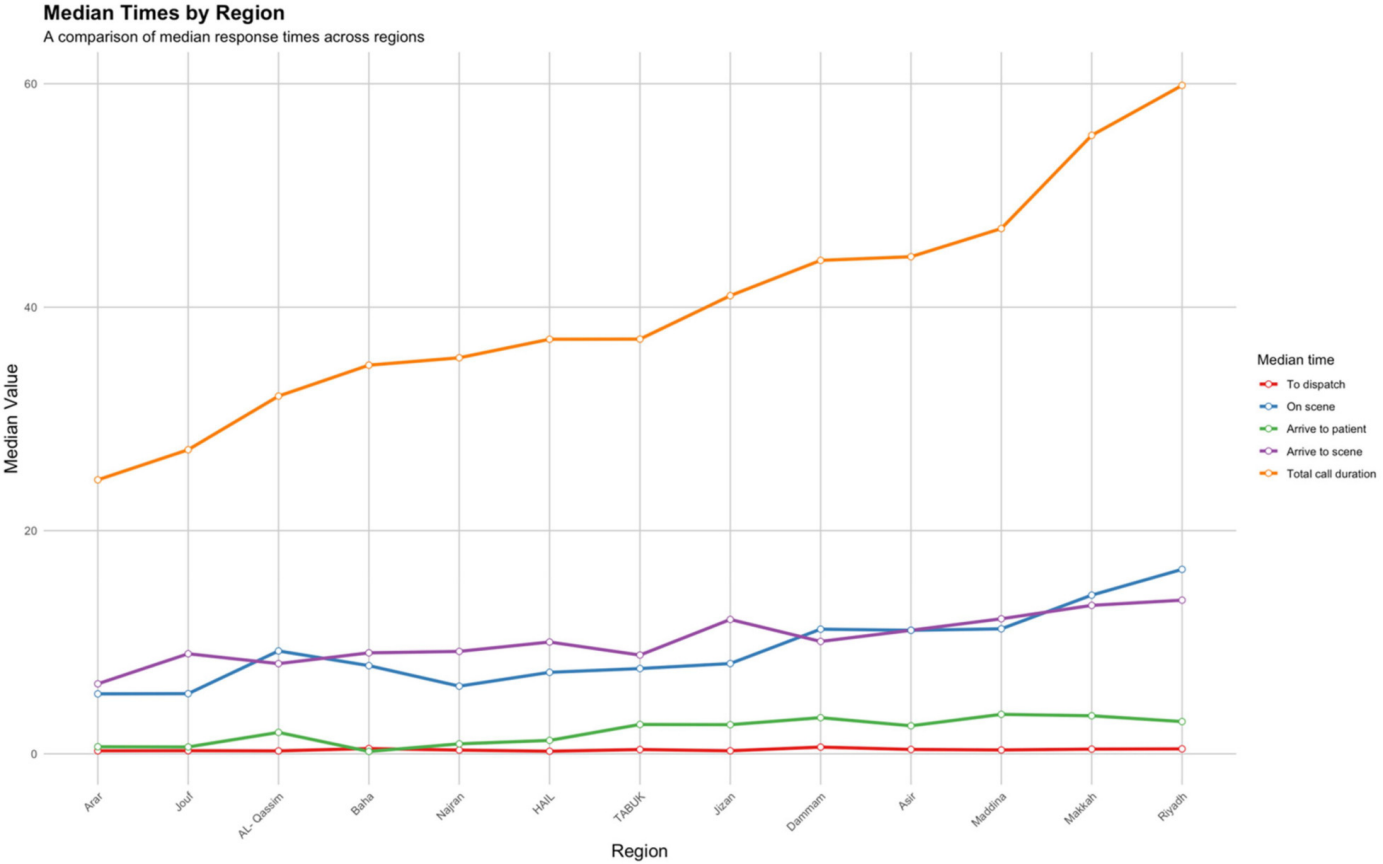
Median EMS response time by province.

Response times varied across different stages of EMS response (Table 3). The median response time was 11.71 min while the median call duration was 48.40 min (IQR: 35.08–65.55). Analysis of the EMS response time by province also showed different response times in all provinces of Saudi Arabia at all stages of EMS response (Figure 6). For response time, Arar had the lowest median time, 6.27 min (IQR: 4.43–8.71), compared to Riyadh, which had the highest median scene time, 13.77 min (IQR: 9.04–20.22) (Figure 6). Among the cities analyzed, Arar also had the lowest median total call duration, at 24.54 min (IQR: 18.73–31.83), while Riyadh had the highest, at 59.85 min (IQR: 44.95–77.40) (Figure 6).

**Table 3:** Summary of emergency medical service response, response times.

Average response time	Time (Min)	
Receiving call to dispatch Crew	1.79	
Dispatch to scene arrival	13.64	
Scene arrival to patient	4.68	
Average scene time	14.96	
Average call duration	53.89	
Median response time	Median	IQR
Receiving call to dispatch	0.39	(0.25–0.82)
Crew dispatch to scene arrival	11.71	(7.45–17.76)
Scene arrival to patient	2.71	(0.59–6.48)
Median scene time	12.35	(7.12–19.48)
Median call duration	48.40	(35.08–65.55)

## 4 Discussion

This national study provides the first comprehensive profile of pediatric EMS use in Saudi Arabia. The analysis showed that adolescents and school-aged children formed the largest proportion of cases, with boys more frequently represented. Although medical emergencies were more common, trauma and injury-related complaints remained among the leading reasons for EMS activation (17), causing a significant public health burden in Saudi Arabia, particularly injuries arising from road traffic crashes (18, 19). A wide range of emergencies was observed, including musculoskeletal, respiratory, and neurological conditions, underscoring the diverse skills and preparedness required of paramedics. Regional differences were also apparent, reflecting both population distribution and variation in EMS demand. Most cases were managed by basic life support crews, highlighting their central role in frontline pediatric emergency care.

Most pediatric EMS transported cases were encountered between 9:00 pm and 1:00 am, with higher frequencies observed during weekends. These peaks likely reflect the cultural context in Saudi Arabia, where families commonly stay up late, particularly on weekends, and children frequently engage in social and recreational activities outside the home. Increased family gatherings, outings, and road travel during these times may elevate the risk of accidents and medical emergencies. The study also found higher case frequencies during school months, particularly in May, September, November, and December. These peaks may be related to increased school-related commuting, participation in sports and outdoor activities (20). These findings align with global and local evidence that pediatric emergencies follow predictable temporal cycles, with the summer season demonstrating the lowest EMS utilization (21, 22), underscoring the importance of aligning ambulance deployment and workforce scheduling with anticipated demand.

The spectrum of pediatric emergencies observed in this study is broadly consistent with national and international literature, where trauma, respiratory, and neurological conditions are among the most frequent drivers of emergency care activations (23–26). In high-income countries such as the United States, trauma consistently accounts for a substantial proportion of pediatric EMS calls, while respiratory illnesses, gastrointestinal conditions, psychiatric emergencies, and seizures are also commonly reported (27–29). Similar trends have been reported in Europe and Australia, although the relative proportions differ according to population demographics and healthcare-seeking behaviours. On the other hane, a comprehensive regional research detailing the patterns of pediatric trauma, highlighting trauma as one of the primary causes of pediatric emergency cases in the Saudi Araiba.

In contrast, studies from low- and middle-income countries, including India and Pakistan, have reported fever, respiratory conditions, and abdominal illnesses as the most common reasons for pediatric emergences (30, 31). The predominance of trauma and injury-related complaints in our findings mirrors patterns seen globally (32), yet the contribution of medical cases such as neurological and musculoskeletal complaints underscores the diverse training and preparedness required of Saudi paramedics when responding to pediatric patients.

These results have significant implications for both public health and EMS practice in Saudi Arabia. The fact that most pediatric cases were managed by BLS crews highlights the importance of strengthening training in key areas such as airway management, trauma care, bleeding control, and other common emergencies. Regular refresher courses and simulation-based training should be practiced helping maintain skills in low-frequency but high-acuity cases. At the system level, the clear temporal trends observed in this study point to the need for flexible ambulance deployment and increased capacity in regions with higher demand. From a public health perspective, EMS data can guide prevention programs targeting injuries, respiratory problems, and poisoning, while also promoting caregiver awareness and encouraging appropriate use of EMS services.

This study has several strengths, including its national scope, large sample size, and comprehensive coverage of pediatric EMS calls, which enhance the generalisability of the findings. Nonetheless, some limitations should be acknowledged. The study relied on prehospital data, which may be affected by documentation errors or missing information, and clinical outcomes after EMS handover were not available. In addition, the descriptive design limits the interpretation of associations, and detailed subgroup analyses were not performed.

### 4.1 Limitations

The study has several limitions, our data only report the prehospital phase as currently the health prehospital care system is not linked to hospital system in which it was imposable to obtain clinical relevance (e.g., admission, mortality, interventions). In addition, this study only limited to one year which prevented any analysis of trends or seasonal variations over time, therefore, future studies should consider multi-year data to enhance temporal insights.

### 4.2 Recommendation

Future research should focus on establishing data linkage between prehospital and hospital systems to enable assessment of key clinical outcomes such as admissions, interventions, morbidity, and mortality. Implementing an integrated electronic medical record pathway would further support comprehensive data sharing. Additionally, analyzing multi-year datasets is essential to capture temporal trends and seasonal variations, while expanding data sources and geographic coverage can enhance the generalizability of findings. Incorporating both quantitative and qualitative operational data in future studies would also provide a more complete understanding of prehospital care performance.

## 5 Conclusion

In conclusion, this study provides the first national profile of pediatric EMS utilization in Saudi Arabia, outlining common emergencies as well as temporal and regional patterns. The findings emphasize the need for enhanced system planning and better integration of EMS data into broader public health efforts. Future research should focus on evaluating patient outcomes and identifying strategies to optimise resource allocation across regions. Strengthening pediatric EMS services remains essential to advancing child health and safety in the Kingdom.

## Data Availability

The raw data supporting the conclusions of this article will be made available by the authors, without undue reservation.
